# Characterization of a Heterojunction Silicon Solar Cell by Means of Impedance Spectroscopy

**DOI:** 10.3390/mi15020184

**Published:** 2024-01-26

**Authors:** Kazybek Aimaganbetov, Darkhan Yerezhep, Mussabek Kishkenebayev, Nikolay Chuchvaga, Nurlan Almas, Serekbol Tokmoldin, Nurlan Tokmoldin

**Affiliations:** 1 Institute of Physics and Technology, Satbayev University, Almaty 050032, Kazakhstan; kazybek012@gmail.com (K.A.); darhan_13@physics.kz (D.Y.); chuchvaga@sci.kz (N.C.); 2 Institute of Energy and Mechanical Engineering, Satbayev University, Almaty 050040, Kazakhstan; m.kishkenebayev@gmail.com; 3 Department of Science and Innovation, Astana IT University, Astana 01000, Kazakhstan; nurlanalmasov@gmail.com; 4 Silica Metals LLP, Almaty 050032, Kazakhstan; stokmoldin@gmail.com; 5 Paul Drude Institute for Solid State Electronics, 10117 Berlin, Germany; 6 Institute of Physics and Astronomy, University of Potsdam, 14476 Potsdam, Germany

**Keywords:** impedance spectroscopy, heterojunction silicon solar cells, carrier lifetime, capacitance

## Abstract

Impedance spectroscopy provides relevant knowledge on the recombination and extraction of photogenerated charge carriers in various types of photovoltaic devices. In particular, this method is of great benefit to the study of crystalline silicon (c-Si)-based solar cells, a market-dominating commercial technology, for example, in terms of the comparison of various types of c-Si devices. This study investigates the dark and light electrophysical characteristics of a heterojunction silicon solar cell fabricated using plasma-enhanced chemical vapor deposition. The measurements are performed at various applied biases, enabling the determination of complex resistance, characteristic time, capacitive response and impurity concentration within the semiconductor junction and to correlate them with the device performance. In addition, the impedance spectra of the studied cell were investigated as a function of temperature. Studies of the frequency and temperature dependences of capacitance do not reveal a significant presence of thermally activated centers of free carrier capture, concomitant with a very small value of the activation energy extracted from an Arrhenius-type analysis. This leads to a conclusion that these centers are likely not impactful on the device operation and efficiency.

## 1. Introduction

Steady improvement in the performance of photovoltaic devices requires an in-depth understanding of their operation and the knowledge of their various properties and characteristics [1,2]. Among the many research techniques, impedance spectroscopy (IS), a method of alternating current (AC) characterization, has been recognized as a valuable tool for the characterization of charge carriers within semiconductor junctions [3]. Although a solar cell is a direct current (DC) device, it possesses a complex impedance that depends on the operating conditions, such as illumination intensity and applied bias. One approach of investigating the solar cell performance under AC conditions includes monitoring the changes in the device impedance imposed by external illumination and bias. In practical terms, these changes may reproduce variations in the device performance during a day [4].

IS measurements are generally performed in a relatively wide frequency in the millihertz (mHz) to megahertz (MHz) range and enable us to obtain information about studied systems consisting of a combination of interphase and bulk processes, such as transport, recombination and interface states, which are relevant for the performance of optoelectronic devices, including solar cells [5,6]. Beyond solar cells, other semiconductor structures and their representation in terms of the equivalent circuit are widely characterized by means of IS to investigate the interface and near-interface properties of semiconductors [7]. The measurement is performed by applying an alternating voltage *V_ac_* to the measured sample and measuring the current response *I_ac_* [8]:

$$V\left(t\right)=V_{0}sin(\omega t)=V_{0}e^{i\omega t}$$

(1) $$I\left(t\right)=I_{0}\sin\left(\omega t+\varphi \right)=I_{0}e^{i\left(\omega t+\varphi \right)}$$

where $V_{0}$ is the amplitude of the voltage signal, $I_{0}$ is the amplitude of the current signal, ω is the angular frequency and φ is the phase. The impedance *Z*(*t*) is given by:

(2) $$Z\left(t\right)=\frac{V(t)}{I(t)}=\frac{V_{0}}{I_{0}}e^{i\varphi }$$

Physically, the impedance is the complex (time-varying or frequency-dependent) resistance of the sample:

(3) $$Z=Z^{\prime }+iZ^{\prime \prime }$$

where *Z′* is the real component and Z″ is the imaginary component of the complex value *Z*.

The experimental method is simple, but the interpretation and analysis of the results often requires detailed models to evaluate the mechanisms involved. In solar cells, it is important to conduct the frequency analysis both in the forward and reverse directions of the p–n junction bias because this enables us to determine the displacement–dependent capacitance. This is one of the previously proposed methods used to obtain information on carrier recombination and lifetime due to the fact that either the displacement or the illumination changes the dynamics of the device, and this change becomes visible through the change in the capacitance [4]. It is ultimately important to identify the mechanisms that determine the operation of photovoltaic cells between the short circuit and open circuit conditions, which is the operating range for power generation.

In this study, we show that IS provides valuable information about the factors determining the photoelectric characteristics of a heterojunction silicon (Si) solar cell at various applied voltages in the dark and under illumination, as well as at different temperatures. This class of devices is an excellent alternative for the manufacture of high-efficiency solar cells and a strong candidate for the worldwide dissemination of photovoltaic technologies at the industrial scale [9,10]. With their history starting in 1941, with the discovery of photovoltaic properties of crystalline Si (c-Si) and the description of the first concepts of photovoltaic devices [11], Si-based solar cells reached an efficiency milestone of 6% in 1954 using diffusive p–n junctions [12]. The first a-Si:H/c-Si heterostructures started to be investigated in 1974 [13,14], and in 1983, the first heterojunction solar cell based on a-Si:H/poly-Si was obtained [15,16], followed by a period of their active investigation [17]. In 1980, Sanyo used Si heterojunctions to achieve an efficiency of 12%, which improved considerably over the years. The decisive technological breakthrough was the creation of a thin buffer layer between the doped amorphous silicon and the wafer to reduce the density of the interface states. Such structures showed efficiencies of up to approximately 14.5%. In particular, due to the buffer layer, record high values for solar cells were achieved. The use of such heterostructures on both sides of the wafer, with front and back buffer layers, has increased the efficiency of the cells up to 18%, further rising upon optimization to above 26% [18].

To this day, many research papers have been devoted to the study of Si photovoltaic devices using IS. Mora-Seró et al. [19] employed the technique to characterize silicon solar cells at various illumination intensities and in the dark. The impedance behavior was examined at various applied biases for every illumination condition. Thus, for varying illumination intensities, a range of cell metrics was obtained as a function of bias voltage, including series and parallel resistances, capacitance, minority carrier lifetime, acceptor impurity density and charge density in the depletion layer. Lee et al. [20] investigated the influence of diborane flow rate during the doping of a p-type solar cell layer as well as the static and dynamic characteristics of the solar cell using IS. The p-layer coordination number of boron atoms was employed to study the p/i interface in thin-film amorphous silicon solar cells. IS measurements showed that the integrated potential and diode idealization factor fluctuate with the diborane flow rate. Bouzidi et al. [21] analyzed the characteristics of a single-crystal silicon solar cell under both dark and light conditions using impedance measurements. By fitting the measured impedance data to an AC-equivalent circuit, the measurements made under illuminated conditions were examined in terms of the electronic behavior. As illumination increases, the angular impedance’s phase shifts from negative to positive values. It exhibits a progressive decline as the frequency increases, culminating in a plateau that signifies its autonomy from illumination at elevated frequencies. Different lighting and dark environments affected AC conductivity differently. According to the authors’ conclusion, the solar cell responds to light impedance very sensitively, indicating that it would be a good fit for photosensor devices. In the paper by Orpella et al. [22], the authors described a novel application of IS to measure the rear point contact (R_base_) of a p-type silicon solar cell. These measurements at high frequencies enable the study of R_base_ because they contain a capacitor consisting of a semiconductor, a dielectric and a metallic structure. This technique is based on fitting the IS measurement results to a physical model that allows the reproduction of the dependence of R_base_ on the carrier injection and substrate doping density. The experimental R_base_ data, which are in the best agreement with the analytical model at a contact radius of 33 μm, show a decrease with increasing bias voltage associated with the carrier injection. Matacena et al. [23] described the fabrication of graphene-based electrical contacts for solar cells. IS was used to characterize the contacts and compared with the traditional gold contact fabrication technique. Using the impedance method, the capacitance-voltage of the graphene–silicon solar cell, as well as the height of the barrier formed at the graphene–silicon interface, were determined. In spite of the relative simplicity of IS as a measurement method, it has not been widely applied to crystalline silicon (c-Si)-based solar cells, especially in modern high-efficiency silicon devices with higher efficiencies. Van Nijen et al. demonstrated the use of IS to characterize laminates of different commercial c-Si PV cells, comparing their capacitance at the point of maximum power and inductance associated with the design of the cell metallization [24]. Shehata et al. applied the technique to c-Si based solar cells with SiO_x_/poly-Si back passivating contacts and an efficiency of 21.25% [25]. They succeeded in extracting the resistive and capacitive components associated with the p^+^–n junction and n^+^–poly-n junction, which cannot be determined using conventional DC measurements. With these parameters, the authors were able to determine the relevant time constants and lifetimes. The results showed the importance of IS in investigating dynamic characteristics of high-efficiency c-Si solar cells. Panigrahi et al. employed IS to analyze the electrical characteristics of solar cells with an a-Si:H/c-Si heterojunction (SHJ) both in the dark and under illumination [26]. According to their findings, the required dispersion parameter for modeling the IS of an SHJ cell indicated a broad distribution of time constants, i.e., a shallow distribution of exponential tailing trap centers in the amorphous layer and/or at the c-Si/a-Si:H interface. Investigation of these electrical parameters as a function of temperature and illumination, and comparison with models of heterojunctions, helped to explain the mechanism of efficiency enhancement in heterojunction cells. 

The investigation of Si solar cells at different temperatures is an important approach for the study of defects and activation characteristics in devices. In a study by Garland et al. [27], the temperature and voltampere dependences of several parameters that describe the performance of a single-crystalline n^+^–p Si solar cell were studied in detail using impedance spectroscopy techniques. A range of existing theoretical models were used to investigate the mechanisms behind the observed temperature/electrical sensitive properties of the cell. The measured cell parameters’ temperature and voltage dependence highlighted the distinct roles of defect-induced charge recombination and minor carrier diffusion. These parameters include the diode’s effective ideality factor; the acceptor concentration in the base; the series, shunt, and recombination resistances; the transition layer capacitance; and the integrated n^+^–p interface potential.

With regards to the analysis of experimental data on interfacial processes in silicon solar cells, a thorough review of numerous mathematical models and temperature dependencies is presented in Reference [28]. The primary focus of the discussion was placed on the analysis of relaxation times and the clarification of resistive and recombination losses in solar cells related to various physical processes involving their resistive and capacitive components. These parameters are primarily described by DC methods, which make it challenging to learn about certain crucial parameters like interfacial diffusion and junction capacitance, as well as the resistive and capacitive elements of a silicon solar cell’s p–p^+^ interface. As a result, comprehensive experimental data combined with a suitable analytical model could be applied in a straightforward fashion to solar cell characterization and combined with conventional photovoltaic testing.

Heterojunction Si cells are a benchmark in the market in terms of performance, so the detailed studies and comparison of their properties, determined via such high-throughput techniques as impedance spectroscopy, may appear fruitful for the development of new solar cell concepts [29]. This raises a question if a direct correlation exists between the solar cell parameters determined via impedance spectroscopy and the device efficiency, which requires a comparison and analysis of earlier works and motivates the current research.

## 2. Materials and Methods

The studied heterojunction Si solar cell was fabricated using an earlier reported procedure [30]. The Si wafers were purchased from Atecom Technology Co., Ltd. (Taipei, Taiwan). Water for the chemical process was distilled using an Electrodeinization System Model MX100 from Martin Water Technologies Inc. (Shanghai, China). Water quality with a resistivity of about ~2 MΩ cm was measured using a Mettler Toledo SevenGo Duo portable conductometer pH/Ion/Cond Meter SG78.

The complete fabrication sequence consisted of several steps. The first stage involved the wet chemical treatment of c-Si n-type wafers to remove surface contaminants and to texturize the wafers [31]. The texturization process of wafers is necessary to reduce their reflectivity and increase their optical absorption. The texturing stage consisted of the following steps:

(1) Degreasing at 65 °C, 1% KOH + 1% H_2_O_2_
(2) Etching with 10% KOH
(3) Texturizing with 1% KOH, + 6% IPA
(4) Degreasing at 65 °C, 4 min, 1% KOH + 1% H_2_O_2_
(5) Metal removal at 20 °C, 3 min, HF + HCl 1:1
(6) Obtaining an oxide layer at 40% HNO_3_
(7) Oxide film removal at 20 °C, 8% HF.

After each step, the plates were rinsed in deionized water twice for two minutes each. The solutions for degreasing, etching, texturing and oxide film were heated to 65 °C, respectively, on a Heidolph MR 3001K magnetic stir plate (Schwabach, Germany). Deionized water, metal removal and oxide film stripping solutions were held at room temperature. All processes were carried out under vigorous and controlled stirring at 400 rpm.

At the next stage, following the removal of the oxide layer and texturing, the Si wafers were sent to a plasma-enhanced chemical vapor deposition (PECVD) chamber for coating of an a-Si:H internal passivation layer using monosilane (SiH_4_) as process gases and amorphous silicon doped with boron and phosphorus impurities using trimethyl borane (B(CH_3_)_3_) and phosphine (PH_3_) as process gases. Next, transparent indium tin oxide (ITO) electrodes were deposited on both sides of the samples using physical vapor deposition (PVD). ITO is a wide bandgap n-type semiconductor with a bandgap width of 3.5–4.3 eV. The ITO films were synthesized via magnetron sputtering (PVD, AK1000 line (Meyer Burger, Germany)) at the base pressure of 5 × 10^−6^ torr and using an indium oxide (90%)/tin oxide (10%) alloy target. The synthesis of the films was carried out at the magnetron power of 2000 W and at the argon flow of 200 cm^3^/s and oxygen flow of 4.8 cm^3^/s at room temperature. 

**Figure 1:**
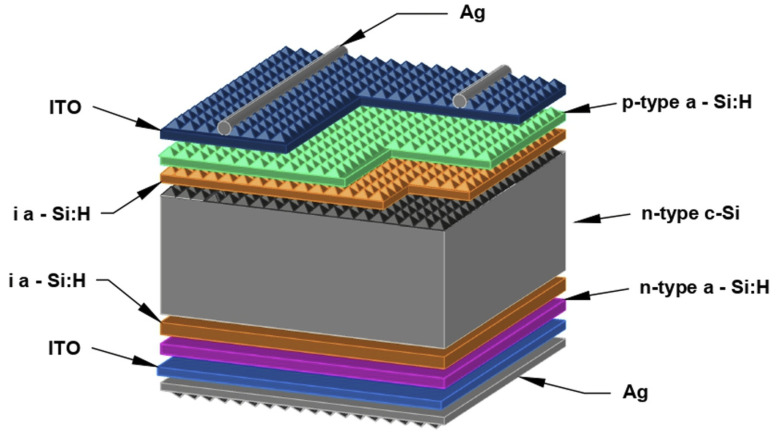
Structure of the fabricated heterojunction silicon solar cell.

Metallization of wafers on the front side was carried out with the help of a metal mesh applied using screen printing with the help of DEK Eclipse Technology (Weymouth, UK). The rear side of the cell was additionally coated with silver (Ag). An illustration of the device structure under study with a top-printed Ag grid is shown in Figure 1. 

**Figure 2:**
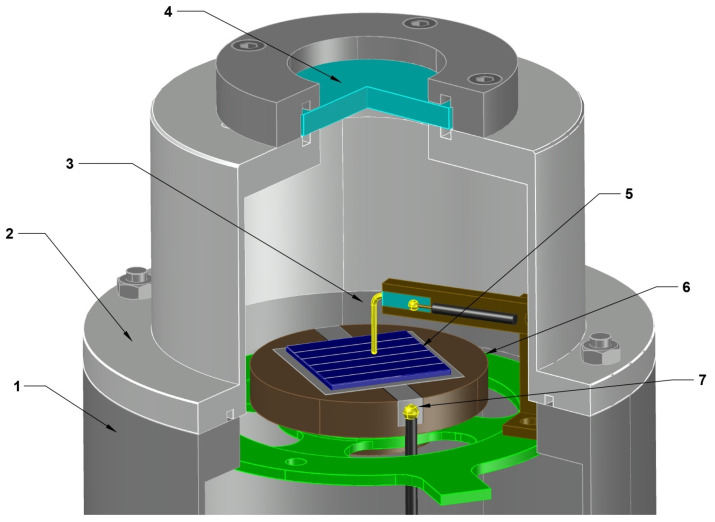
Three-dimensional drawing of the low-temperature chamber employed for the impedance measurements: 1—microcryogenic machine body; 2—cell cover; 3—electrical contacts; 4—viewing window; 5—test sample; 6—substrate; 7—electrical contacts.

The I-V curves were obtained using a PV Measurements, Inc. model IV-16L measuring system. The measuring system produces 1 solar illumination (AM1.5) and maintains a constant temperature of 25 °C, avoiding heating due to illumination. The surface morphology of the wafers was investigated using a JEOL JSM-6490LA (Tokyo, Japan) scanning electron microscope with an accelerating voltage of 30 kW. The impedance measurements were performed using an HF2 Impedance spectroscope (Zurich, Switzerland) in the frequency range from 1 Hz to 1 MHz with an AC amplitude of 25 mV in the dark and under illumination (~1 sun). The applied voltage was varied from −0.1 V to 0.7 V at room temperature. The measurements were performed in a homemade measuring cell based on a Cryomech Model ST15 Cryostat microcryogenic machine specially designed to study the electrophysical properties of semiconductor devices at low temperatures [32,33]. The developed experimental cell allowed us to carry out electrophysical measurements in different temperature ranges. Figure 2 depicts a schematic structure of the measuring cell.

## 3. Results

**Figure 3:**
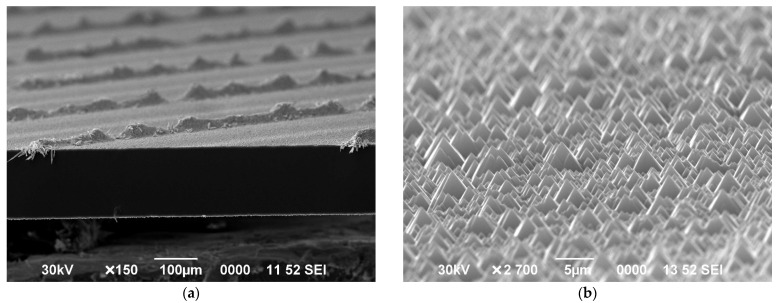
SEM images of the sample surface: (**a**) general view of the solar cell (side view); (**b**) with pyramids with an average height of 5 μm (side view).

Surface images of the initial and textured solar cell, obtained by means of scanning electron microscopy (SEM), are shown in Figure 3 (side view). The size of the resulting pyramids is approximately 5 μm. It is similarly noted that pyramids with dimensions of 2–8 μm are usually characterized by a reflection coefficient of 12–15% at wavelengths of incident light 400–700 nm [34].

**Figure 4:**
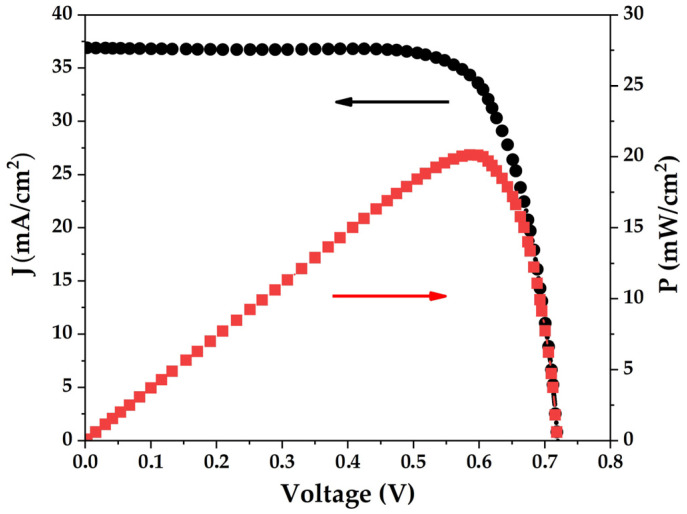
Illuminated I-V characteristic of the Si solar cell under study. The black circles show the dependence of current density on applied voltage (indicated by the black arrow), whereas the red squares show the dependence of power density on applied voltage (indicated by the red arrow).

The light I-V characteristic of the studied solar cell is shown in Figure 4 and the performance parameters including power conversion efficiency (PCE, *η*), fill-factor (*FF*), open-circuit voltage (*V_OC_*) and short-circuit current density (*J_SC_*) are given in Table 1. The performance is lower than that of record Si-based solar cells, but breaks the 20% efficiency hurdle.

The impedance measurements were performed under vacuum to avoid measurement-induced degradation; however, device handling following fabrication, before and after the I-V measurements, unavoidably involved exposure to an ambient atmosphere. Upon the analysis of IS measurements on a semiconductor device, interpretation of the capacitive contribution is important as the primary step when determining a correct model that describes carrier transport and recombination. In this regard, device characterization in the dark is useful for studying their behavior in the absence of photogenerated carriers. A typical example is the well-known Mott–Schottky analysis. This procedure is carried out in the dark and covers a range of reverse and moderate forward biases, when the device capacitance is determined by the width of the depletion region [35].

**Figure 5:**
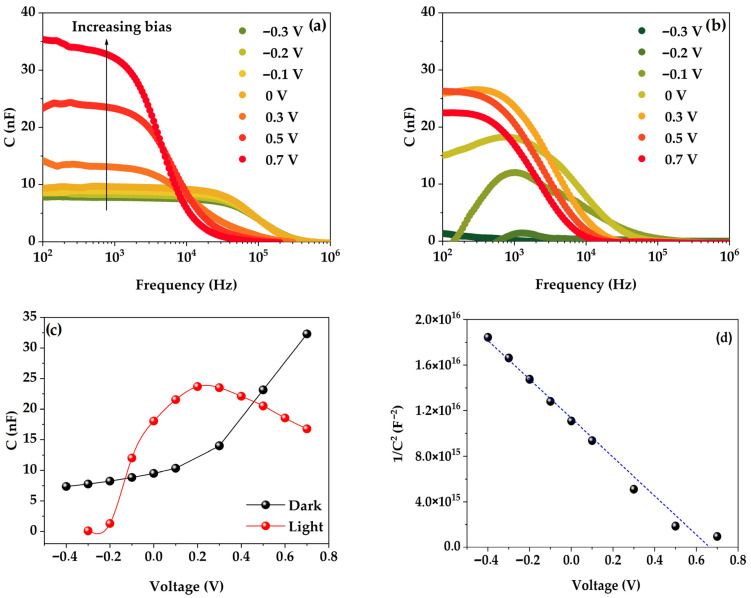
Capacitance characteristics of the solar cell under study: (**a**) *C*-*f* in the dark; (**b**) *C*-*f* under illumination; (**c**) *C*-*V* at 1 kHz in the dark (black) and under illumination (red); (**d**) 1/*C*^2^ at 1 kHz versus applied voltage in the dark.

First, both frequency and voltage-dependent capacitance characteristics were tested to confirm that the diffusion capacitance was clearly distinguishable from the depletion capacitance in the studied bias range. The light and dark capacitance-frequency (*C*-*f*) spectra (Figure 5a,b) were measured in the range from 100 Hz to 1 MHz. Surprisingly, device capacitance measured under illumination was measured to be lower than that in the dark for all applied biases, except for a limited range from −0.1 to 0.3 V. This could be due to a strong contribution of negative diffusion capacitance, which was earlier reported to be significant under illumination in Si-based solar cells and was attributed to the impact of nonideal metal–semiconductor contacts [4]. Capacitance-voltage (*C*-*V*) characteristics were measured at a frequency of 1 kHz to determine the built-in voltage (*V_bi_*) and the doping concentration (*N_D_*) using the following equation [2,19,20,36,37,38]:

(4) $$\frac{1}{C^{2}}=\frac{2(V_{bi}-V)}{A^{2}q\varepsilon \varepsilon_{0}N_{D}}$$

where *C* is the capacitance of the depletion layer, *A* is the device area, *q* is the elementary charge, *ε* is the semiconductor dielectric constant, *ε*_0_ is the dielectric constant of vacuum and *N_D_* is concentration of the alloying donor (D) impurity in the wafer. By applying Equation (4) [25], we take into account the low thickness of the intrinsic a-Si layers and a much lower doping density of the n-type Si wafer compared to the p-doped a-Si emitter. 

**Table 1:** Correlation of the silicon solar cell parameters obtained from the impedance measurements in this study and in the literature: *N_D_*—doping density, *V_bi_*—built-in voltage, *τ_relax,dark,Isc_*—relaxation time, *τ_light,Voc_*—carrier lifetime, *η*—power conversion efficiency, *FF*—fill-factor, *J_SC_*—short-circuit current density, *V_OC_*—open-circuit voltage.

Structure	*N_D_*, cm^−3^	*V_bi_*, V	*τ_relax,dark,Isc_*, μs	*τ_light,Voc_*, μs	*η*, %	*FF*	*J_SC_*, mA/cm^2^	*V_OC_*, V	Reference
n^+^pp^+^ (p-type Si) (B)	-	0.96	-	1	9.0	0.65	24.2	0.577	[6]
npp^+^ (p-type Si) (A)	-	0.76	-	10	9.2	0.61	27.7	0.546	[6]
PEDOT:PSS(rGO) (n-type Si)	2.48 × 10^18^	0.34	0.017	-	0.02	0.21	40.5	0.2	[38]
npp^+^ (BP ‘‘Saturn’’) (p-type Si)	3.92 × 10^16^	0.71	-	20	12.0	0.70	35.0	0.480	[19]
n^+^p (A) (p-type Si)	1.1 × 10^16^	0.69	~200	-	17.0	0.70	38.1	0.617	[2]
p^+^nn^+^ (n-type Si)	6.42 × 10^16^	0.67	374	83	20.1	0.759	36.7	0.721	This work

**Figure 6:**
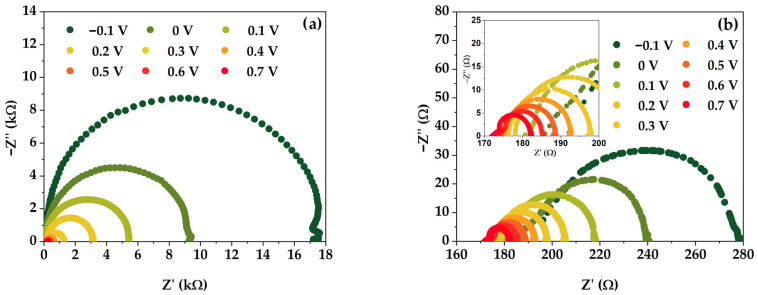
Complex impedance characteristics of the heterojunction Si solar cell under study (representing the functional dependence *Z″*(*ω*) = *f*(*Z′*(*ω*)): (**a**) in the dark; (**b**) under illumination (1 sun).

Examining Figure 5c,d, one can notice that a characteristic behavior of a depletion layer capacitance is already apparent at inverse applied voltages [39]. The built-in potential is calculated from a linear fit of 1/*C*^2^ versus applied voltage, yielding the built-in voltage of *V_bi_* = 0.67 V and the impurity concentration of *N_D_* = 6.42 × 10^16^ cm^−3^ for the studied solar cell. The impedance spectra of the solar cell measured at different *V_app_* (ranging from −0.1 V to 0.7 V) in the dark and under illumination (1 sun) are shown in Figure 6. 

The IS enables the characterization of the carrier recombination in a photovoltaic cell by means of a characteristic time constant, often referred to as the carrier lifetime, *τ*. This time can be measured by means of intensive photomodulation, performed via illuminating the sample under study while measuring the impedance signal [40]:

(5) $$\tau =R_{rec}\times C_{\mu }$$

where $R_{rec}$ is the recombination resistance and $C_{\mu }$ is the chemical capacitance. This characteristic time corresponds to the angular frequency at the apex of the complex impedance arc *Z″*(*ω*) = *f*(*Z′*(*ω*)):

(6) $$\omega =\frac{1}{\tau }$$

**Figure 7:**
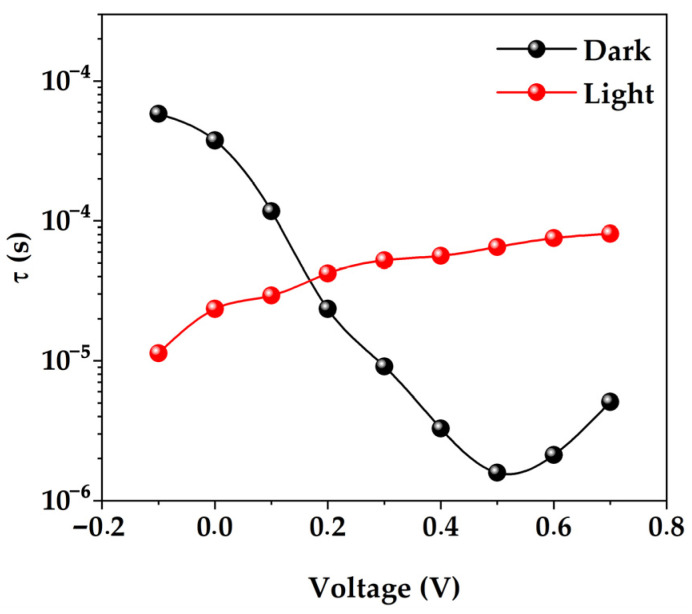
Dependence of the characteristic relaxation time and the effective lifetime of minority carriers calculated from impedance measurements on the applied voltage.

According to Equation (6), *τ* can be used to calculate the effective lifetime [2,8,19,41], provided that the barrier layer capacitance is known from measurements shown in Figure 6. The characteristic time obtained from the frequency corresponding to the apex of the complex impedance arc at different biases is shown in Figure 7.

**Figure 8:**
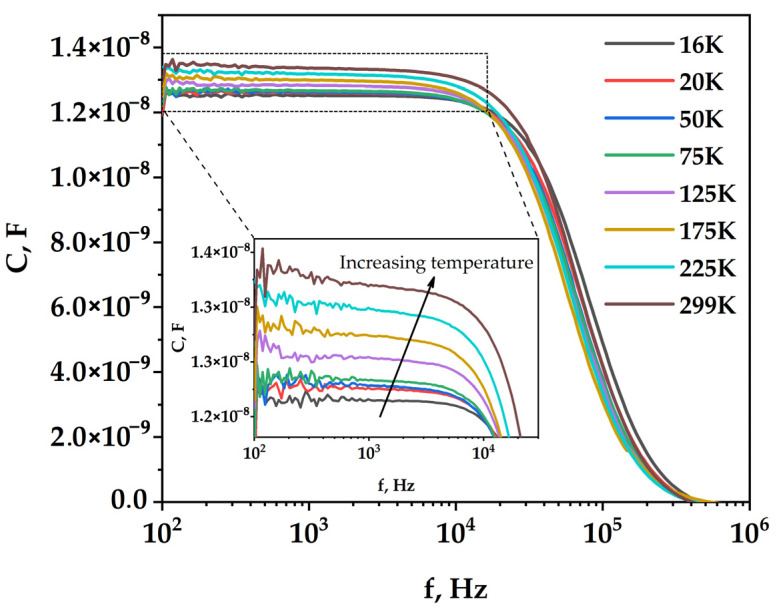
Dark C-f measurements of a silicon solar cell at a constant bias of 0 V in the frequency range from 100 Hz to 1 MHz as a function of temperature (16–300 K).

As evidenced by Table 1, illumination intensity dependent studies on silicon solar cells have been performed rather routinely [2,6,19,38]. At the same time, according to our knowledge, the impact of temperature on the IS measurements of such devices has been explored to a very limited extent. Such measurements provide valuable information on the quality of the studied junctions and enable us to detect and quantify defect states that may have a detrimental impact on performance [42,43,44,45,46,47,48,49]. In order to evaluate the quality of the studied junction, dark measurements of the *C-f* characteristics were carried out in the temperature range from 16 to 300 K (Figure 8) in the range from 100 Hz to 1 MHz. For all temperatures, a characteristic shape of the capacitance spectrum is observed, with a plateau at lower frequencies followed by a smooth decrease at frequencies above 10 kHz. It can be seen that the capacitance increases with temperature in the low-frequency range. This is expected because more carriers are thermally activated at higher temperatures due to an increase in the emission rate, resulting in an increase in the effective bulk charge density and capacitance [43]. In addition, there is an obvious shift in the frequency of the capacitance drop-off with decreasing temperature, which can be attributed to a simultaneous impact of varying resistance and capacitance determining the *RC* relaxation time of the circuit.

**Figure 9:**
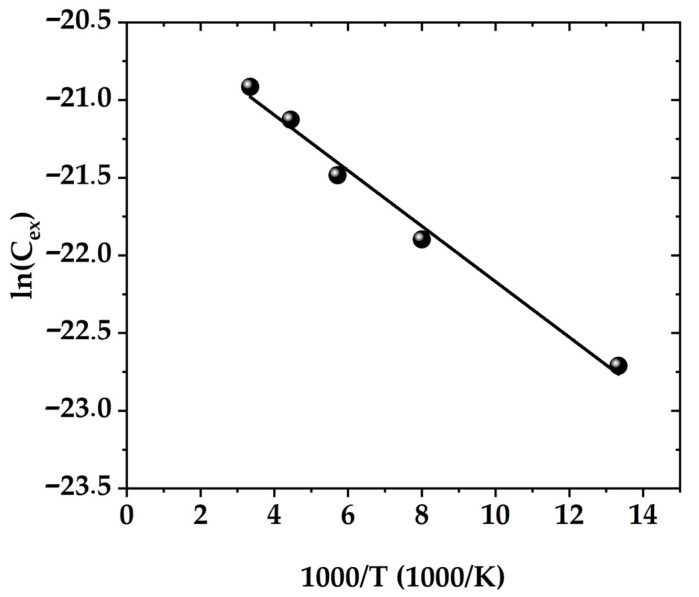
Arrhenius plot for the extraction of *E_a_* for thermally activated centers of free carrier capture.

Assuming that the capacitive signal is the sum of the barrier and diffusion capacitance associated with thermally activated carriers, the activation energy *E_a_* equal to 15 meV (Figure 9) was calculated from the temperature dependence of the thermally activated contribution to the diffusion capacitance *C_exc_ = C − C_16_*_K_:

(7) $$C_{exc}=C_{0}e^{\left(\frac{-E_{a}}{kT}\right)}$$

where *E_a_* is the effective activation energy and *C_0_* is a prefactor. When the total capacitance is used for the same Arrhenius-type analysis, the extracted value of *E_a_* is even lower. 

Based on the conducted analysis, we conclude that in the studied device, the charges in the thermally activated capture centers do not play a significant role in the capacitance increase with temperature.

## 4. Conclusions

Impedance spectroscopy measurements were carried out on a heterojunction silicon solar cell to investigate the properties of the barrier junction and to study the minority carrier recombination. The dark and light characteristics at different bias voltages, including complex resistance, characteristic time, capacitive response and impurity concentration of the studied devices were extracted and analyzed. Although IS can be considered as an indirect method to the study of electrical properties of semiconductor devices, the ability of this technique to probe their frequency response, including the frequency and voltage-dependent capacitance characteristics, provides a means of characterizing both dielectric and injection-modulated properties of the semiconductor junctions. Determination of capacitance-frequency spectra from measured impedance data on the basis of either a simple parallel or series R-C circuit provides an opportunity for a standardized and unambiguous analysis of studied devices. Herein, an effort was made to perform such a unified capacitance-based analysis complemented with temperature-dependent impedance spectroscopy measurements on a Si heterojunction cell. A strong correlation was observed between the characteristic carrier lifetimes obtained using impedance spectroscopy and the device performance. In addition, the temperature-dependent capacitance analysis revealed a rather insignificant role of thermally activated centers of carrier capture in affecting the device diffusion capacitance. Our findings highlight the effectiveness of this simple experimental technique to obtain operationally relevant junction characteristics and help predict the device performance. This underscores the growing importance of detailed capacitance-based characterization of both commercially relevant and emerging photovoltaic devices for their continued optimization and improvement.

## Data Availability

The raw data supporting the conclusions of this article will be made available by the authors on request.
